# Risk perception and adherence to preventive behaviours related to the COVID-19 pandemic: a community-based study applying the health belief model

**DOI:** 10.1192/bjo.2021.954

**Published:** 2021-07-13

**Authors:** Aziz Kamran, Khatereh Isazadehfar, Heshmatolah Heydari, Ramin Nasimi Doost Azgomi, Mahdi Naeim

**Affiliations:** School of Medicine and Allied Medical Sciences, Ardabil University of Medical Sciences, Iran; School of Medicine and Allied Medical Sciences, Social Determinants of Health Research Center, Ardabil University of Medical Sciences, Iran; Department of Nursing, School of Nursing and Midwifery, Social Determinants of Health Research Center, Lorestan University of Medical Sciences, Iran; French Institute of Research and Higher Education (IFRESINT), France; Pharmaceutical Sciences Research Center, Ardabil University of Medical Sciences, Iran; Social Determinants of Health Research Center, Ardabil University of Medical Sciences, Iran

**Keywords:** Perception, COVID-19, behaviour, adherence

## Abstract

**Background:**

Coronavirus disease 2019 (COVID-19), now a global pandemic, is a new, highly contagious, and preventable disease that has caused many deaths across the world. Correct understanding of the risks and following health instructions are among the most important self-care parameters.

**Aims:**

To assess people's perception of the risks and their adherence to recommended preventive behaviours regarding COVID-19 infection.

**Method:**

This descriptive–analytical study was conducted with 1861 people residing in Ardabil province in 2020. The data were collected electronically and included four elements: demographic details; health belief model (HBM) constructs (perceived sensitivity, perceived severity and perceived benefits); beliefs about the effectiveness of disease prevention strategies; and complying with health behaviours. The data was analysed using SPSS-21 software.

**Results:**

Significant differences were found in the mean scores for beliefs about the effectiveness of preventative measures, the constructs of the health belief model, and compliance with preventive behaviours relating to the participants’ gender, age, marital status and level of education. Beliefs and intention to stay at home, collectively predicted 54.7% of the variance in preventive behaviours.

**Conclusions:**

Although a majority of participants had positive attitudes towards the effectiveness of preventive measures and adhered to them, some people who were not adherent with these healthy behaviours could be key participants in the next wave of the disease.

## Background

Coronavirus disease 2019 (COVID-19) is an infectious disease caused by a novel severe acute respiratory syndrome (SARS)-related coronavirus which was first reported on 31 December 2019.^1,2^ After a rapid spread affecting most countries across the world, the World Health Organization (WHO) declared the disease a pandemic on 11 March 2020.^3^ Common symptoms include fever, cough, and a shortness of breath.^4,5^ Although symptoms are mild in most patients, the disease is sometimes associated with the failure of vital organs such as lungs, the heart and kidneys.^6^ The disease is typically transmitted through infected respiratory droplets following coughing or sneezing and can be prevented by regular hand washing, restricting social contact and observing health guidelines.^7,8^

Up until 12 May 2020, the number of people infected with COVID-19 had reached more than 4 million, and the death toll had hit over 2000 worldwide.^9,10^ The first case of COVID-19 infection in Iran was observed in Qom city on Tuesday, 18 February 2020.^11,12^ Afterwards, the disease started to spread rapidly and the mortality rate raced upwards exponentially. To 12 May 2020, the number of confirmed cases of patients with COVID-19 in Iran was 107 603, and the number of deaths was 6640.^9,10^ Reported reasons for a high death toll or the number of infections include poor health literacy perceived threat anxiety, depression enabling factors, perceived benefits level or urbanisation, its social and religious norms social norms, perceived benefits of quarantine and perceived risk of the disease.^13–18^

From the earliest days after detecting the disease in Iran, health officials banned the public from attending group meetings and encouraged them to stay at home. However, these warnings seemed ineffective, and some people continued to gather across the community. We believe this is the first community-based study with a large study sample that has examined adherence to recommended measures in Iran.

## Health belief model

We used the health belief model (HBM) to theoretically examine behavioural predictors.^19–21^ This model has six structural components including perceived sensitivity, severity, benefits, barriers, practical guidelines and self-efficacy. As reported in recent studies, this model can be used to provide self-care recommendations.^22,23^ According to this model, individuals engage in self-care behaviours when they believe that the consequences of not adhering to such behaviours are serious. In this way, one is encouraged to comply with preventive behaviours.^24–26^ Considering the high capacity of this model to predict people's health-related behaviours and their perceptions about health recommendations, we decided to conduct a study to assess risk perception and the rate of adherence to preventive recommendations regarding COVID-19 infection in Iranian people.

For this purpose, we used the HBM to assess factors determining the rate of social adherence to preventive measures. In this way, it is possible to identify and implement appropriate interventions to prevent the spread of the disease across the community.

## Method

This descriptive–analytical study was conducted with 1960 people in Ardabil province between April and May 2020. A total of 1861 questionnaires were analysed after removing incomplete ones. The data was collected electronically by submitting the questionnaires via the social media that were popular across the province.

The inclusion criteria were age 20–70 years, ability to read the questions and respond with answers, owning either a smartphone, a laptop or a computer, having access to popular social media in Iran (i.e. Telegram, WhatsApp, Baleh, and Gap). Exclusion criteria were having an academic background in a health-related fields (medicine, nursing, pharmacology, etc.) and not completing the entire questionnaire.

At the top of the questionnaire, declarations were provided regarding the goals of the study, as well as approvals from the Research Center for Social Sciences and the Ethic Committee of Ardabil University of Medical Sciences. There was no need for the participants to provide personal information such as names and national IDs. We explained the objectives of the study sufficiently in the first section of the electronic instructions; participating in the study was voluntarily and on the basis that they provided their full consent.

The data was collected using a four-part questionnaire. The first part included demographic variables. The second part was related to risk perception assessed through three structural constructs of the health belief model; perceived sensitivity (four items), perceived risk (four items) and perceived benefits (two items). These parameters were determined by a five-option Likert scale with responses from ‘strongly agree’ to ‘strongly disagree’. The ranges of scores on the perceived sensitivity, severity and benefits constructs were 4–20, 4–20 and 2–10, respectively.

The third part of the questionnaire was related to beliefs about the effectiveness of health recommendations (nine items). Finally, the last part of the survey assessed behaviours for preventing COVID-19 infection (nine items).

The sections on ‘beliefs on the effectiveness of health recommendations’ and ‘compliance with recommended behaviours’ were assessed based on a Likert scale that was scored from one to seven. A score of one designated the lowest belief and compliance rates, and a score of seven indicated the highest belief and compliance rates. Therefore, the scores for these two sections ranged from 9 to 63.

The validity of the questionnaire was confirmed using face and content validity (i.e. content validity index and content validity ratio indicators). The reliability of the instrument was confirmed based on Cronbach's alpha coefficients of 0.87 and 0.85 for the belief and behaviour sections, respectively.

The data were analysed using SPSS statistical software (version 21). Numerical variables were reported as mean and standard error, and qualitative variables were expressed by frequencies. For comparing the mean scores of the variables, either sample Student's *t*-test (two independent groups) or one-way ANOVA (more than two independent groups) were used. Correlations between the variables were assessed using the Pearson correlation test. Multiple linear regression tests were used to evaluate the predictive value of risk perception and beliefs on the variance of social health behaviours.

## Results

Our results showed that 996 (53.5%) of the participants were female. Furthermore, 1363 (73.2%) were married, and 877 (47.1%) had an undergraduate-associate degree. Also, 638 (34.3%) of the participants were housewives and 807 (43.4%) were in the age group 30 to 40 years.

Overall, 970 (52.1%) of the participants reported that they had acquired their information only from social media. Another 722 (38.8%) people declared that the SMS messages sent by the Ministry of Health had weak or very weak effects on their behaviours. Finally, 1434 (77.1%) and 353 (19%) of the participants announced that they agreed and completely agreed, respectively, with the slogan of ‘we stay at home’.

The results showed that 15.6% (290) of participants perceived COVID-19 as a simple and similar to flu and colds, and 14.6% (272) agreed that ‘The disease is dangerous only for the elderly and diabetics and cardiovascular patients’, 7.3% (135) participants believed ‘I am not in danger’. The mean score of perceived sensitivity, severity and benefits was 15.9 (s.e. = 2.2), 16.5 (s.e. = 2.2) and 8.7 (s.e. = 1.1), respectively (Table 1).

**Table 1:** Descriptive statistics for the health belief model constructs

Constructs, item	Strongly disagree	Disagree	No comment	Agree	Strongly agree	Mean (s.e.)
Perceived sensitivity						15.9 (2.2)
The disease is simple and similar to flu and colds	720 (38.7)	516 (27.7)	335 (18)	213 (11.4)	77 (4.1)	
The disease is dangerous only for the elderly and diabetics and cardiovascular patients	656 (35.2)	810 (43.5)	123 (6.6)	181 (9.7)	91 (4.9)	
I am not in danger and my immune system is strong	610 (32.8)	903 (48.5)	213 (11.4)	91 (4.9)	44 (2.4)	
Health advice doesn't matter, and it doesn't affect my daily behaviour	343 (18.4)	1453 (78.1)	23 (1.2)	23 (1.2)	19 (1.0)	
Perceived severity						16.5 (2.0)
I feel it is necessary to reduce communication and personal contact, and I must avoid infected people	19 (1)	54 (2.9)	12 (0.6)	265 (14.2)	1511 (81.2)	
Corona morbidity and mortality news is important to me and I follow it	31 (1.7)	24 (1.3)	130 (7.0)	810 (43.5)	866 (46.5)	
I am worried about the behaviour of others and the statistics of the disease in the future	22 (1.2)	14 (0.8)	70 (3.8)	604 (32.5)	1151 (61.8)	
The facilities and capacity of the health system, such as doctors and nurses, are limited and I am worried about the future	125 (6.7)	24 (1.3)	146 (7.8)	627 (33.7)	939 (50.5)	
Perceived benefits						8.7 (1.1)
Adherence to the principles of prevention has important effect on the course of COVID-19 disease	49 (2.6)	58 (3.1)	82 (4.4)	1161 (62.4)	511 (27.5)	
I stay home so that fewer people get infected and the epidemic of the disease is controlled sooner	26 (1.4)	13 (0.7)	35 (1.9)	353 (19.0)	1434 (77.1)	

COVID, coronavirus disease 2019.

The results showed that the mean scores for behaviour in women were significantly higher than men and scores for attitudes towards behaviour were significantly higher in men than women (*P* < 0.001). Also, the mean scores for behaviour and attitude in people over 50 years (*P* < 0.001) and housewives were significantly higher than those for other age groups and occupational groups (*P* < 0.001); farmers and ranchers reported the lowest mean score for behaviour and attitude. The results showed that the mean score for behaviour and attitude towards behaviour was significantly higher in married participants than others (Table 2).

**Table 2:** Comparison of scores for attitude and behaviour by gender, age groups, occupations and marital status

	Behaviour total		Attitude total	
Variables	Mean (s.d.)	*P*	Mean (s.d.)	*P*
Gender				
Male	54.16 (10.0)	<0.001	55.5 (9.4)	<0.001
Female	56.92 (8.2)		57.5 (7.6)	
Age group, years				
20–30	56.4 (8.6)	0.01	55.3 (9.4)	0.003
31–40	57.1 (7.6)		56.2 (8.4)	
41–50	55.9 (9.5)		55.2 (9.9)	
More than 50	57.4 (8.6)		56.8 (8.2)	
Occupation				
Student	55.7 (9.0)	<0.001	54.7 (9.0)	<0.001
Employee	56.8 (7.8)		55.9 (7.8)	
Shopkeeper	55.9 (9.0)		54.2 (9.0)	
House keeping	57.6 (7.9)		56.9 (7.9)	
Agricultural and livestock jobs	49.9 (16.2)		48.8 (16.2)	
Driver	56.6 (7.4)		54.5 (7.4)	
Marital status				
Single	55.78 (8.5)	0.03	54.64 (9.0)	0.02
Married	56.90 (8.6)		55.97 (9.2)	
Divorced/widowed	57.77 (6.4)		55.86 (8.1)	

Significant differences were not seen in perceived sensitivity and severity among men and women but the mean score for perceived benefits in women was significantly higher than men. People in employment had significantly higher mean scores for perceived sensitivity, severity and benefits. Also, the mean scores for perceived sensitivity in the age group 31–40 years and perceived severity and benefits in people over 50 years were significantly higher than scores for other age groups (*P* < 0.001). The results showed that the mean scores for perceived sensitivity were significantly higher in single participants and the mean scores for perceived severity and benefits were significantly higher in married participants than those who were single or divorced/widowed (*P* < 0.001) (Table 3).

**Table 3:** Comparison of scores for the health belief model constructs by gender, age groups, jobs and marital status

	Perceived sensitivity		Perceived severity		Perceived benefits	
Variables	Mean (s.d.)	*P*	Mean (s.d.)	*P*	Mean (s.d.)	*P*
Gender						
Male	15.8 (2.3)	0.1	16.4 (2.0)	0.3	8.6 (1.2)	<0.001
Female	16.0 (2.2)		16.5 (2.0)		8.9 (0.9)	
Age group, years						
20–30	15.95 (2.2)	0.08	16.4 (2.1)	0.008	8.7 (1.2)	<0.001
31–40	16.1 (2.1)		16.6 (2)		8.8 (1)	
41–50	15.7 (2.4)		16.6 (1.9)		8.9 (0.7)	
Over than 50	15.7 (2.2)		16.8 (1.7)		8.9 (0.9)	
Occupation						
Student	15.99 (2.2)	0.13	16.3 (2.2)	0.18	8.60 (1.4)	0.009
Employee	16.11 (2)		16.68 (1.9)		8.88 (0.9)	
Shopkeeper	15.86 (2.3)		16.56 (2)		8.74 (1)	
House keeping	15.97 (2.3)		16.53 (2)		8.84 (1)	
Agricultural and livestock jobs	15.26 (2.2)		16.13 (2.4)		8.66 (1.5)	
Driver	15.59 (2.6)		16.52 (2.2)		8.68 (1.2)	
Marital status						
Single	16.13 (2.1)	0.02	16.41 (2.2)	0.04	8.5 (1.4)	<0.001
Married	15.93 (2.2)		16.59 (1.9)		8.86 (0.9)	
Divorced/Widowed	15.19 (2.9)		15.93 (2.4)		8.47 (1.3)	

Result showed that the majority of people believed that the perceived sensitivity, perceived severity and perceived benefits recommended in the media were effective in preventing COVID-19 disease. However, for 18% (287 people) of participants wearing gloves outside the home, for 3.7% (70 people) of participants wearing masks in contact with patients, for 10.2% (189 people) of participants hand washing with disinfectants and for 3% (56 people) of participants washing hands with soap and water were considered to be very ineffective, ineffective or somewhat ineffective (rating of 1–3). Given that belief in the effectiveness of each item was measured from one (minimum, very ineffective) to seven (maximum, very effective), a rating of four can be considered as having doubt about effectiveness, which was selected by a significant number of participants (Table 4).

**Table 4:** Descriptive statistics for attitude towards the effectiveness of the recommendations to prevent coronavirus disease 2019 (COVID-19)

Item	Participants perceived rate of effectiveness of recommendations (1 = very ineffective)						
	1	2	3	4	5	6	7
Wearing gloves outside the home	110 (5.9)	65 (3.5)	112 (6.0)	209 (11.2)	272 (14.6)	148 (8.0)	945 (50.8)
Wearing a mask outside the home	134 (7.2)	68 (3.7)	133 (7.1)	232 (12.5)	267 (14.3)	184 (9.9)	843 (45.3)
Wearing masks in contact with patients and people with suspected COVID-19	51 (2.7)	13 (0.7)	6 (0.3)	22 (1.2)	35 (1.9)	43 (2.3)	1690 (90.8)
Stay at home	49 (2.6)	2 (0.1)	11 (0.6)	25 (1.3)	50 (2.7)	99 (5.3)	1625 (87.3)
Lack of attendance at family and religious ceremonies	57 (3.1)	20 (1.1)	4 (0.2)	15 (0.8)	39 (2.1)	79 (4.2)	1647 (88.5)
Minimise public presence such as purchases and banking	53 (2.8)	266 (14.3)	8 (0.4)	25 (1.3)	65 (3.5)	153 (8.2)	1551 (83.3)
Hand washing after contact with outdoor equipment with disinfectant	80 (4.3)	29 (1.6)	80 (4.3)	154 (8.3)	205 (11.0)	151 (8.1)	1162 (62.4)
Wash hands after contact with outdoor appliances with soap and water	48 (2.6)	4 (0.2)	4 (0.2)	21 (1.1)	47 (2.5)	100 (5.4)	1637 (88.0)
Disinfect personal items such as keys, mobile phones and cars	56 (3.0)	4 (0.2)	18 (1.0)	34 (1.8)	80 (4.3)	145 (7.8)	1524 (81.9)

Results showed that the majority of participants adhered to the recommended behaviours about preventing catching COVID-19 disease. But 18.1% (337 people) reported never, rarely or very infrequently (ratings of 1–3) wearing gloves outside the home; 21.6% (402 people) wearing masks outside the home; 6.1% (114 people) wearing masks in contact with patients with COVID-19 and people with suspected COVID-19; 12.8% (239 people) washing their hands with disinfectants containing 70% alcohol; and 3.9% (73 people) washing their hands with soap and water reported the same behaviour adherence of never, rarely or very infrequently. Given that the adherence to the recommended behaviours for each item was measured from one (minimum) to seven (maximum), a rating of four can be considered as incomplete adherence to the recommendations; which was selected by a significant number of participants (Table 5).

**Table 5:** Descriptive statistics for behaviour adherence towards the recommendations to prevent coronavirus disease 2019 (COVID-19)

Behaviours	Participants rate of adherence to preventative measures (1 = never)						
	1	2	3	4	5	6	7
Wearing gloves outside the home	169 (9.1)	82 (4.4)	86 (4.6)	158 (8.5)	235 (12.6)	192 (10.3)	939 (50.5)
Wearing a mask outside the home	188 (10.1)	90 (4.8)	124 (6.7)	177 (9.5)	208 (11.2)	182 (9.8)	892 (47.9)
Wearing masks in contact with patients and people with suspected COVID-19	99 (5.3)	5 (0.3)	10 (0.5)	22 (1.2)	33 (1.8)	53 (2.8)	1639 (88.1)
Stay at home	30 (1.6)	43 (2.3)	17 (0.9)	49 (2.6)	140 (7.5)	192 (10.3)	1390 (74.7)
Lack of attendance at family and religious ceremonies	66 (3.5)	5 (0.3)	15 (0.8)	29 (1.6)	48 (2.6)	136 (7.3)	1557 (83.7)
Minimise public presence such as purchases and banking	27 (1.5)	26 (1.4)	17 (0.9)	48 (2.6)	107 (5.7)	210 (11.3)	1426 (76.6)
Hand washing after contact with outdoor equipment with disinfectant	108 (5.8)	62 (3.3)	69 (3.7)	117 (6.3)	211 (11.3)	189 (10.2)	1105 (59.4)
Wash hands after contact with outdoor appliances with soap and water	46 (2.5)	6 (0.3)	21 (1.1)	20 (1.1)	69 (3.7)	130 (7.0)	1569 (84.3)
Disinfect personal items such as keys, mobile phones and cars	39 (2.1)	31 (1.7)	31 (1.7)	57 (3.1)	101 (5.4)	152 (8.2)	1450 (77.9)

Analysis of the results in Table 5 showed that the total attitude score and staying at home can alter behaviour in a significant way. According to our results, 54.7% of the total variance about behaviour can be explained though these two variables (Table 6).

**Table 6:** Multiple linear regression analysis about predictor of coronavirus disease 2019 (COVID-19) preventive behaviours

	*B*	s.e.	Standardised beta	*t*	*P*	*R*	*R*^2^
Total attitude score	0.748	0.018	0.696	41.599	<0.001	0.74	0.547
Stay at home	1.414	0.228	0.104	6.212	<0.001		

## Discussion

### Main findings

This study aimed to explain perception of risk and adherence to preventive health recommendations regarding COVID-19 infection in the Iranian population. Based on the structural constructs of the health belief model, the perceived sensitivity, severity and benefits had desirable levels in the studied population. Despite this, some people refused to participate in the required health behaviours that are needed in order to prevent and control the disease. Moreover, this study shows that the ‘attitude towards the disease’ and ‘the intention to stay at home’ were the most important predictors of health behaviour adherence in the community.

### Interpretation of our findings

Our findings showed that women followed advice about how to behaviour more than men and had a better attitude than men regarding the prevention of COVID-19. This was despite the fact that men generally experience a more severe form of the disease and have a higher risk of mortality.^27^ In this study, attitude and perceived benefits were more positive in women than in men. In contrast, the findings of Abdulhafez et al showed that men and women had the same levels of knowledge and the same attitudes regarding COVID-19.^28^ Likewise, a study on the H1N1 pandemic ( a novel influenza A) revealed more avoidance behaviour in women compared with men.^29^ These discrepancies between genders may be related to the personality differences of men and women as females are generally much more sensitive and influential than males in perceiving their surroundings.^29^ Therefore, it is necessary to use appropriate sensitisation methods to help men to better understand the benefits of adhering to the COVID-19 preventive measures.

In this study, knowledge about the disease and behaviour differed by age group. People over the age of 50 years had better perceptions and avoidance behaviours towards the disease. One of the reasons for this finding may be a misunderstanding that had spread on social media that only older people were at risk of this disease.^30^ Contrary to the findings of this study, other reports have shown that younger people possess better knowledge and attitudes towards the disease and towards preventive measures. This may be related to social, cultural, political and economic differences among different communities. According to the findings of Abdulhafez et al, older age, lower literacy and income, as well as living in rural areas were associated with inferior knowledge about the disease.^28^

In this study, housewives had both good perceptions and adopted positive behaviours towards COVID. On the other hand, farmers and drivers had little knowledge and negative behaviours. This observation of ours was similar to that of another study in Egypt noting better knowledge and attitude towards the disease and preventive measures in people living in cities compared with those living in rural areas, or people with an income of less than 5000 Egyptian Pounds per month.^28^ It seems that access to social media, having free time to search information sources, educational level and social culture can be determining factors regarding knowledge and behaviour towards COVID-19 infection. Usually, people living in rural areas have limited access to scientific facilities, poor studying habits, as well as lower income and less free time than residents in urban regions. Therefore, it is necessary to improve awareness and attitudes towards the disease by implementing appropriate programmes, especially across low-income communities.^28^

Our study's findings showed that sensitisation toward the disease was well established in our participants who had seen themselves at risk of the infection. In fact, there was fear of the disease among the population. The perceived sensitivity was higher in middle-aged people (30 to 40 years) than others. This may reflect a sense of curiosity, activism, and better access to social media and the internet in this age group. Becoming alert to the threats and consequences of the disease can augment the rate of adherence with preventive health behaviours. Furthermore, we noticed that perceived severity scores were higher in individuals over 50 years than those in other age groups. This may be explained by the fact that elderly people and people with underlying diseases may experience a more severe form of the disease, which ultimately leads to a higher mortality rate in these groups. Of note, our participants were fully aware of this fact, which was in parallel with the findings of another study indicating perceived severity as an important predictor of adherence to health behaviours.^31^ Yet another study showed that perceived severity was positively associated with negative emotions and higher utilisation of mobile phones leading to more appropriate health behaviours to contain the COVID-19 pandemic.^32^

Our findings showed that perceived sensitivity scores were higher in single people. On the other hand, perceived severity and benefits scores were higher in married individuals. This can be attributed to the more free time and better access to data resources in single people and to the fact that married individuals are more attached to their martial life.^32^ In the same way, married individuals may have a greater concerntowards environmental threats. We also found that the perceived benefit scores were higher in elderly people than young individuals. One explanation might be that a higher perceived severity and fear of disease complications would lead elderly people to better understood the advantages of preventive behaviours (i.e. regular hand washing, applying face masks and keeping to social distancing).

The findings of the present study indicated homogeneous perceived sensitivity and severity comparing women and men; nevertheless, perceived benefit scores were higher in women than in men. This was in parallel with another study suggesting that parameters such as moving to a new neighbourhood, having good knowledge and awareness about the disease, and the fear of the disease can encourage people to observe appropriate health behaviours.^33^

In this study, the majority of the participants noted that they obtained the information they required about the disease and about preventive measures through social media. Still, some of them had low adherence to appropriate health behaviours. In line with the findings of this study, it has been reported that most people acquire their knowledge about the disease through social media and the internet.^23^ In the another study, despite good knowledge in the general population about transmission patterns and common symptoms of COVID-19 disease, they misunderstood preventive actions because of confusing information circulating on social media.^34^ As people may carry out incorrect behaviours such as unauthorised consumption of antibiotics, it is important to improve people's knowledge and awareness about the disease and preventive actions through formal and valid websites and mass media, as well as education from knowledgeable and trusted individuals such as doctors, nurses and other health staff.^34^

The results of our study show that most of the participants abided by the required disease prevention behaviours. People's beliefs about the effectiveness of the treatments they would recieve upon visiting a hospital can be among the factors influencing the rate of behavioural compliance.^35^ Therefore, providing accurate and reliable information along with the necessary personal protective equipment (PPE) can increase the rate of public adherence to health behaviours amid this pandemic.^36^ Although a large portion of our participants followed appropriate health behaviours, there were also people who, despite good perceived sensitivity and severity, and high awareness, ignored these behavioural protocols. One of the most important reasons for this phenomenon may be people getting used to the situation over time. Other influencing factors include economic problems and either a shortage in or high prices of PPE.^37^

In this study, it was shown that two variables: attitude towards the disease and tendency to stay at home, predicted more than 50% of the variance in COVID-19 preventive social health behaviours. This was in line with the findings of two studies in China showing the efficiency of lockdown to significantly reduce the incidence of and mortality from COVID-19 infection.^38,39^ According to the guidelines of the WHO, one important way to control and manage the disease is to maintain social distancing and avoid attending group meetings.^40^ The public staying at home requires national, social and personal determination. In order to encourage people to stay at home, infrastructure must first be provided, and schools and universities should be closed and education continued through online courses. The online education itself requires appropriate infrastructure and the preparation of teachers and students. Furthermore, organisations and agencies that provide essential services should continue to operate with minimal staff. The livelihood needs of the public should be provided, and broadcasting agencies should provide entertainment programmes. On the other hand, some people who may not have a regular income (i.e. workers who are hired on a daily/temporary basis) must be supported by governments and non-governmental organisations. In this way, it is necessary to pave the road for the public to keep to social distancing and adhere to required preventative health behaviours.

### Limitations

One of the limitations of this study was that it was undertaken electronically using social media to keep the researchers and participants safe. Therefore, controlling for cofounding variables and inclusion/exclusion criteria may have been somehow limited. By including a relatively large sample size, we tried to overcome this limitation to some extent. Another limitation of this study was the unavoidable exclusion of socially deprived groups who did not own smartphones or access to social media. This should be considered when generalising our data to other communities.

### Implications

Based on the structural constructs of the health belief model, we found optimal levels of perceived sensitivity, severity and benefits among our participants. Despite this, some people still did not comply with appropriate preventive health behaviours to contain the COVID-19 pandemic. The most important factors predicting social healthy behaviours were individual's attitude towards the disease and the their intention to stay at home. Complying with healthy behaviours was lower in middle-aged and younger people than in elderly people, which may have its root in a myth stating that only older people are affected by the disease. It is therefore recommended that the public are provided with reliable, accurate and scientific information sources by creating valid social media and virtual network updates. Our study shows that although the majority of the study participants followed disease prevention measures, there were also individuals who did not adhere to these measures. Therefore, it is necessary to identify the barriers leading these people to not to comply with the appropriate health behaviours and resolve their issues with them. Two variables, attitude towards the disease and the intention to stay at home, predicted more than 50% of the variance observed in the adherence to preventive health behaviours across the community. Finally, providing necessary infrastructure can encourage people to stay at home and comply with social distancing protocols amid the pandemic.

## Data Availability

The original data are available on request to the corresponding author, after the manuscript published.
