# Risk perception and adherence to preventive behaviours related to the COVID-19 pandemic: a community-based study applying the health belief model – CORRIGENDUM

**DOI:** 10.1192/bjo.2021.980

**Published:** 2021-11-16

**Authors:** Aziz Kamran, Khatereh Isazadehfar, Heshmatolah Heydari, Ramin Nasimi Doost Azgomi, Mahdi Naeim

**Keywords:** Perception, COVID-19, behaviour, adherence, erratum

This article^1^ contains the wrong affiliation for Ramin Nasimi Doost Azgomi. The correct affiliation is: Traditional Medicine and Hydrotherapy Research Center, Ardabil University of Medical Sciences, Ardabil, Iran.

The publishers apologise for the omission.
